# Incretin Effect in Women with Former Gestational Diabetes within a Short Period after Delivery

**DOI:** 10.1155/2012/247392

**Published:** 2012-04-19

**Authors:** G. Pacini, A. Tura, Y. Winhofer, A. Kautzky-Willer

**Affiliations:** ^1^Metabolic Unit, Institute of Biomedical Engineering, National Research Council, 35127 Padova, Italy; ^2^Department of Internal Medicine III, Medical University of Vienna, 1090 Vienna, Austria

## Abstract

*Background and Aims*. Women with former gestational diabetes (fGDM) are characterized by impaired beta-cell function (BC). Incretin hormones contribute to insulin secretion after oral administration of glucose. We aimed to assess the possible role of incretins on altered insulin release in fGDM. *Materials and Methods*. We studied 104 fGDM women within 6 months after delivery and 35 healthy women after normal pregnancy (CNT) with a 75 g oral (OGTT) and a 0.33 g/kg intravenous (IVGTT) glucose test, both lasting 3 h. The ratio of suprabasal areas under the concentration curves for glucose (dAUC_GL_) and C-peptide (dAUC_CP_) evaluated BC during OGTT (BC_OG_) and IVGTT (BC_IV_). Incretin effect was computed in all fGDM and in fGDM with normal tolerance (fGDM_NGT_) and with impaired glucose regulation (fGDM_IGR_). *Results*. dAUC_GL_ of fGDM was higher (*P* < 0.0001) than CNT for both tests; while dAUC_CP_ were not different. BC_OG_ and BC_IV_ were lower in fGDM versus CNT (1.42 ± 0.17nmol_CP_/mmol_GLUC_ versus 2.53 ± 0.61, *P* = 0.015 and 0.41 ± 0.03 versus 0.68 ± 0.10, *P* = 0.0006, respectively). IE in CNT (66 ± 4 %) was not different from that of all fGDM (59 ± 3) and fGDM_NGT_ (60 ± 3), but higher than that of fGDM_IGR_ (52 ± 6; *P* = 0.03). IE normalized to BMI was 2.77 ± 0.19 % m^2^/kg in CNT, higher than that of fGDM_IGR_ (1.75 ± 0.21; *P* = 0.02) and also of fGDM_NGT  _(2.33 ± 0.11; *P* = 0.038). *Conclusion*. Compromised IE characterizes fGDM_IGR_. In both fGDM categories, regardless their glucose tolerance, IE normalized to BMI was reduced, signifying an intrinsic characteristic of fGDM. Therefore, the diminished IE of fGDM seems to reflect an early abnormality of the general beta-cell dysfunction in the progression toward type 2 diabetes.

## 1. Introduction

The incretin effect is the potentiation of the glucose-mediated insulin secretion due to the gut hormones, namely, glucose-dependent insulinotropic polypeptide (GIP) and glucagon-like peptide-1 (GLP-1). They are released by the intestine in the blood stream after oral glucose load, such as during an oral glucose tolerance test or a meal [1]. The incretin effect has been demonstrated by comparing insulin concentration during oral and intravenous glucose administration that yielded the same peripheral glucose concentration; however, insulin concentration was significantly higher after the oral load. Since this happened despite the same glucose levels than with the intravenous load [2], the difference in insulin concentration was ascribed to the effect of the incretin hormones. In type 2 diabetic patients, a reduction of this effect has been reported [3, 4] and pharmacological agents have been developed to restore this effect, important for maintaining a good glucose homeostasis [5].

Women with former gestational diabetes (fGDM) exhibit markedly increased risk for the later development of type 2 diabetes and related complications. They often present metabolic abnormalities in insulin sensitivity compared to control subjects, but even greater differences have been observed for beta-cell function. In a previous study on fGDM, we have assessed beta-cell function by the analysis of two independent tests, intravenous (IVGTT) and oral (OGTT) glucose tolerance tests and found that the impairment in beta-cell function was observable only with OGTT [6]. We hypothesize that the incretin effect may play a pivotal role in the subtle derangement of beta-cell function observed in fGDM.

Therefore, the aim of this study was to evaluate the incretin effect in a larger group of fGDM, who underwent both IVGTT and OGTT immediately after partum. They were also divided according to their glucose tolerance to evaluate if it plays some role on the possible changes of the incretin effect in fGDM.

## 2. Materials and Methods

A total of 104 fGDM were studied within 6 months after delivery and compared to 35 healthy women after normal pregnancy (CNT).  Table 1 shows the main characteristics of the two groups; body mass index (BMI) of fGDM was borderline higher, but in general still in the overweight range. Every subject randomly underwent a 75 g OGTT with sampling at 10, 20, 30, 60, 90, 120, 150, 180 min and a 0.33 g/kg frequently sampled intravenous glucose test (IVGTT) with insulin (0.03 IU/kg, Humulin R; Eli Lilly, Indianapolis, IN, USA) intravenous infusion at time 20 for 5 min. Less than 3 weeks elapsed between the two tests, without any diet or habit changes in between. Both tests lasted 3 h; glucose and C-peptide were determined in duplicate by commercially available radioimmunoassay kits with an interassay coefficient of variation <5%. Further details on the performance of the two tests were reported previously [7, 8].

Out of 104 fGDM, 77 resulted with normal glucose tolerance (fGDM_NGT_) according to the OGTT criteria of the American Diabetes Association, 6 with type 2 diabetes and 21 with impaired glucose regulation (fGDM_IGR_), which included both impaired fasting glucose and impaired glucose tolerance. All CNT exhibited normal glucose tolerance.

For the purpose of this study, we calculated the area under the concentration curves (AUC) for glucose (AUC_GL_) and C-peptide (AUC_CP_) for 3 h in both tests, by using the trapezoidal rule. The suprabasal, dynamic AUC (dAUC) were computed by subtracting from the AUC the basal area (i.e., fasting value × 180 min). AUC_CP_ represents the absolute insulin release at the beta-cell level. The beta-cell function describes the ability of glucose to stimulate insulin release from the beta-cell. Beta-cell function during OGTT (BC_OG_) and that during IVGTT (BC_IV_) were calculated according to the respective ratio dAUC_CP_/dAUC_GL_ in both tests: units nmol_CP_/mmol_GLUC_. The incretin action occurs only during an oral administration of glucose; thus, it can be estimated by subtracting the glucose stimulated secretion during the IVGTT from that evaluated during the OGTT: the formula 100 × (BC_OG_ − BC_IV_)/BC_OG_ yields therefore the percent incretin effect [3]. This approach has been already successfully exploited in previous studies [9, 10].

In order to evaluate whether different glucose tolerance within the fGDM plays a role in the assessment of beta-cell function and incretin effect, these parameters were also computed in the single subgroups of normotolerant and impaired metabolism.

Data are expressed as mean ± SE; means have been compared with the Student's *t*-test.

## 3. Results

Fasting and total AUC glucose in both tests were markedly higher in fGDM (Table 1); fasting C-peptide was not different, while the dynamic C-peptide response to glucose stimulation only tended to be higher in fGDM, but only with borderline significance. Suprabasal AUC of glucose (dAUC_GL_), which represents the main stimulus to the secretory response of the beta-cell, was higher in fGDM than that of CNT for both tests (338 ± 20 mmol/L 3 h versus 189 ± 21, *P* = 0.0001 for OGTT;  206 ± 13  versus 119 ± 16, *P* = 0.0002 for IVGTT). Dynamic insulin secretion, dAUC_CP_ was not different between fGDM and CNT in both tests, but much higher (*P* < 0.00001) during OGTT (285 ± 9 nmol/L 3 h versus 257 ± 15, *P* = 0.13 fGDM versus CNT for OGTT;  61 ± 3  versus 55 ± 5, *P* = 0.36 for IVGTT).

Beta-cell function and incretin effect are shown in Table 2, where fGDM was also divided into fGDM_NGT_ and fGDM_IGR_, the latter presenting with higher BMI. From these subgroups the 6 type 2 diabetics were excluded, since their small number allowed no statistical power for any possible comparison. BC_OG_ was markedly higher than BC_IV_ in both fGDM and CNT (*P* = 0.003); both BC_OG_ and BC_IV_ were lower in fGDM (all together) compared to CNT; and were lower in fGDM_IGR_ compared to fGDM_NGT_.

When comparing the normotolerant fGDM_NGT_ to CNT, both BC_OG_ and BC_IV_ were not different, while beta-cell functions of fGDM_IGR_ were significantly lower than those of CNT. Incretin effect resulted similar between fGDM all together and CNT; that of fGDM_NGT_ was not different from that of CNT (*P* value ranging in both cases 0.2–0.8); while that of fGDM_IGR_ was significantly lower than that of women with normal pregnancy (Table 2). When incretin effect was normalized to BMI, to take into account that the main determinant of IGR was the increased BMI, the differences in the incretin effect were even more substantial. The value for CNT was 2.77 ± 0.19% m^2^/kg, still higher than that of fGDM_IGR_ (1.75 ± 0.21; *P* = 0.019), but also the difference with that of fGDM_NGT_ (2.33 ± 0.11) became significant (*P* = 0.038). Incretin effect normalized to BMI was 2.21 ± 0.10; *P* = 0.006  versus CNT).

## 4. Discussion

Within a short period after partum, beta-cell function, evaluated both with the oral and with the intravenous glucose tests, was reduced in a general population of overweight women who exhibited gestational diabetes mellitus during pregnancy [7]. Indeed, in both tests, fGDM exhibited C-peptide release only slightly increased despite markedly higher glucose. Results of this study show that incretins do not play a fundamental role in this observed reduced beta-cell function characterizing fGDM: in fact, the surrogate index of incretin effect used here did not differ from the same index evaluated in a population of healthy women, who had a normal pregnancy, studied within the same period after partum. In both CNT and fGDM, the beta-cell function obtained with the OGTT was much higher than that with IVGTT, showing that incretins have a potent effect in both groups. The elevated beta-cell response during OGTT could mask possible significant differences in the beta-cell sensitivity to glucose between fGDM and CNT; however, no difference is also highlighted by the IVGTT, which provides the evaluation independent on incretins.

Our results on beta-cell function seem to be in contrast with a previous study [11], where it is reported an unchanged beta-cell function in fGDM. However, in that study, the authors used the 30 min insulinogenic index with insulin, which is known to be not fully reliable [12], while the present investigation exploited C-peptide evaluated during the whole 3 h duration of the test: a more reliable figure of the incretin-mediated sensitivity of the beta-cell to the glucose stimulation. To quantify this process, we have used the percent (normalized) difference of the beta-cell function between the tests, which gauges the relative contribution of the incretins in fostering glucose-dependent insulin release. We have used the beta-cell function instead of the simple AUCs as previously done [3, 9, 10], because the OGTT and the IVGTT did not yield isoglycemic patterns; thus, we had to normalize the insulin release to glucose with the ratios dAUC_CP_/dAUC_GL_. The incretin effect we estimated is a kind of general measurement and cannot discriminate between the real effect of the incretins and the possible reduction in fGDM of the incretins production [13]. In fact, our measurement is an indirect surrogate, since we have not measured the incretin hormones concentration. This is the main weakness of our study. Having for instance the pattern of GLP-1, we could apply another straightforward method [14] that quantifies the direct incretin action, independently on the actual hormone secretion. Some investigators found a mildly reduced GLP-1 response to oral glucose only during the first 30 min, while the entire 2 h GLP-1 AUC did not differ from that of the control subjects [13]. This was substantiated by other studies that reported no differences in the secretion of incretin hormones after oral glucose ingestion in women with a history of gestational diabetes [11]. Therefore, we are quite confident that the incretin effect we observed in our study is prevalently due to the action of incretins in sensitizing the beta-cell.

A recent report [10] showed that incretin effect is reduced with obesity, which is known to be characterized by insulin resistance and reduced glucose tolerance. Another study related incretin effect to various degrees of glucose intolerance [15]. These findings in a general population were then verified in our fGDM, who were divided into normo- and impaired tolerant. To the best of our knowledge, this is a novelty of our study. We have found a clear reduction in the incretin effect in fGDM with IGR. Incretin effect was instead similar between former GDM with NGT and CNT. The reason may be in the fact that IGR have a slightly higher BMI, though not reaching overt obesity yet. This fact corroborates more our point, since it is known that obesity is accompanied by hyperinsulinemia before the onset of overt diabetes. Thus the circumstance that fGDM_IGR_ still presents reduced beta-cell sensitivity to glucose, despite higher BMI, appears to be an intrinsic defect of this population. In fact, when the incretin effect was normalized to BMI, fGDM_NGT_ too had a lower index, despite similar BMI than CNT. Since the majority of fGDM return normal after delivery, in general, we can claim that a reduced incretin effect likely remains a characteristic of this condition.

In conclusion, normotolerant women with previous gestational diabetes exhibit an incretin effect similar to that of healthy women, who had a normal pregnancy. Compromised incretin effect, proper of obese and type 2 diabetic subjects, characterizes instead fGDM with impaired glucose tolerance, probably related to their, though slightly, augmented body weight. When the incretin effect was evaluated after normalization to BMI, it resulted in reduced in both categories, giving the impression of an intrinsic characteristic of fGDM, regardless of their glucose tolerance. The diminished incretin effect of fGDM seems therefore to reflect an early abnormality of the general beta-cell dysfunction in the progression toward type 2 diabetes.

## Figures and Tables

**Table 1:** Main characteristics and area under the concentration curve (AUC) of glucose and C-peptide, in the two tests, for women with former gestational diabetes (fGDM) and women who had normal pregnancy (CNT).

	fGDM	CNT	*P* value
*N*	104	35	
Age (time of the study) (years)	33.5 ± 0.5	31.8 ± 0.9	0.076
Body mass index (kg/m^2^)	27.3 ± 0.5	25.3 ± 1.0	0.048
Fasting glucose (mmol/L)	5.08 ± 0.08	4.62 ± 0.06	0.0008
Fasting insulin (pmol/L)	60 ± 4	57 ± 5	0.668
Fasting C-peptide (pmol/L)	646 ± 31	582 ± 31	0.258
AUC glucose (OGTT) (mol/L 3 h)	1.26 ± 0.03	1.02 ± 0.02	0.00001
AUC C-peptide (OGTT) (nmol/L 3 h)	402 ± 13	353 ± 19	0.048
AUC glucose (IVGTT) (mol/L 3 h)	0.98 ± 0.02	0.85 ± 0.02	0.0001
AUC C-peptide (IVGTT) (nmol/L 3 h)	150 ± 8	122 ± 9	0.055

**Table 2:** Beta-cell function from OGTT (BC_OG_) and IVGTT (BC_IV_) and incretin effect (IE) in all fGDM and in the two subgroups of fGDM characterized by normal glucose tolerance (fGDM_NGT_) and impaired glucose metabolism (fGDM_IGR_), compared to women who had normal pregnancy (CNT).

	fGDM_IGR_	fGDM_NGT_	*P* ^ a^	CNT	*P* ^ b^	all fGDM	*P* ^ c^
*N*	21	77		35		104	
BMI	30.2 ± 1.0	26.2 ± 0.5	0.001	27.3 ± 0.5	0.001	25.3 ± 1.0	0.048
BC_OG_	0.67 ± 0.06	1.70 ± 0.21	0.014	2.53 ± 0.61	0.023	1.42 ± 0.17	0.016
BC_IV_	0.29 ± 0.17	0.47 ± 0.04	0.011	0.61 ± 0.10	0.020	0.41 ± 0.03	0.012
IE	51.5 ± 5.8	60.0 ± 2.8	0.171	65.8 ± 3.6	0.030	58.9 ± 2.5	0.146

BMI: body mass index (kg/m^2^); units for BC_OG_ and BC_IV_: nmol_CP_/mmol_GLUC_; for IE: %; all fGDM include also 6 fGDM type 2 diabetic women; *P* values: ^a^fGDM_IGR_ versus fGDM_NGT_; ^b^fGDM_IGR_ versus CNT; ^c^all fGDM versus CNT.
